# Barriers for kangaroo mother care (KMC) acceptance, and practices in southern Ethiopia: a model for scaling up uptake and adherence using qualitative study

**DOI:** 10.1186/s12884-020-03409-6

**Published:** 2021-01-07

**Authors:** Selamawit Mengesha Bilal, Henok Tadele, Teshome Abuka Abebo, Birkneh Tilahun Tadesse, Mekonnen Muleta, Fitsum W/Gebriel, Akalewold Alemayehu, Yusuf Haji, Dejene Hailu Kassa, Ayalew Astatkie, Anteneh Asefa, Million Teshome, Aknaw Kawza, Shemels Wangoro, Thomas Brune, Nalini Singhal, Bogale Worku, Khalid Aziz

**Affiliations:** 1 College of Medicine and Health Sciences, Faculty of Health Sciences, School of Public Health, Hawassa University, Hawassa, Ethiopia; 2 Department of Paediatrics, College of Medicine and Health Sciences, Faculty of Medicine, Hawassa University, Hawassa, Ethiopia; 3 Department of Pediatrics and Child Health, College of Health Sciences, School of Medicine, Addis Ababa University, Addis Ababa, Ethiopia; 4 Independent Consultant, Addis Ababa, Ethiopia; 5 Department of Obstetrics and Gynaecology, College of Medicine and Health Sciences, Faculty of Medicine, Hawassa University, Hawassa, Ethiopia; 6 South Nations and Nationalities Regional Health Bureau Head, Hawassa, Ethiopia; 7 South Nations and Nationalities Regional Health Bureau, Maternal and Child Health Director, Hawassa, Ethiopia; 8 Sachs’ Children and Youth Hospital, Stockholm, Sweden; 9 Paediatrics, University of Calgary, Calgary, Canada; 10 School of Medicine, Addis Ababa University, Ethiopian Paediatrics Society, Addis Ababa, Ethiopia; 11 Paediatrics, University of Alberta, Edmonton, Canada

**Keywords:** Kangaroo mother care, Preterm, Low birth weight, Formative research, Implementation model, Low-income country

## Abstract

**Background:**

Globally, approximately 15 million babies are born preterm every year. Complications of prematurity are the leading cause of under-five mortality. There is overwhelming evidence from low, middle, and high-income countries supporting kangaroo mother care (KMC) as an effective strategy to prevent mortality in both preterm and low birth weight (LBW) babies. However, implementation and scale-up of KMC remains a challenge, especially in lowincome countries such as Ethiopia. This formative research study, part of a broader KMC implementation project in Southern Ethiopia, aimed to identify the barriers to KMC implementation and to devise a refined model to deliver KMC across the facility to community continuum.

**Methods:**

A formative research study was conducted in Southern Ethiopia using a qualitative explorative approach that involved both health service providers and community members. Twenty-fourin-depth interviewsand 14 focus group discussions were carried out with 144study participants. The study applied a grounded theory approach to identify,examine, analyse and extract emerging themes, and subsequently develop a model for KMC implementation.

**Results:**

Barriers to KMC practice included gaps in KMC knowledge, attitude and practices among parents of preterm and LBW babies;socioeconomic, cultural and structural factors; thecommunity’s beliefs and valueswith respect to preterm and LBW babies;health professionals’ acceptance of KMC as well as their motivation to implement practices; and shortage of supplies in health facilities.

**Conclusions:**

Our study suggests a comprehensive approach with systematic interventions and support at maternal, family, community, facility and health care provider levels. We propose an implementation model that addresses this community to facility continuum.

**Supplementary Information:**

The online version contains supplementary material available at 10.1186/s12884-020-03409-6.

## Contributions to the literature

- This study adds new insights into the barriers to and challenges of kangaroo mother care (KMC) practice and implementation.
- This study strongly supports existing evidence by recommending evidence-informed steps to scale-up KMC.
- A model has been developed to provide further guidance for researchers, programmers, policy makers and health workers with scaling up KMC.

## Background

Annually, around 15 million children are born preterm worldwide, and complications that arise from prematurity are the leading causes of under-five mortality [1]. Neonatal mortality in According to the 2016 EDHS report only 13% weighed less than 2.5 kg at birth regardless of gestational age, which was only based on 14% of recorded birth weight information. Among the 86% unknown birth weight information, mothers’ subjective estimations were considered, and accordingly 16% of births were very small and 10% are smaller than average [2]. In a similar report, children reported to be small or very small are more likely to die than average or larger at birth. In Ethiopia childhood mortality has declined substantially since 2000 however the change in Neonatal mortality is not significant as the change in other child mortality which remained high and was 29 per 1000 live births.

The Every Newborn Action Plan was endorsed and launched by the World Health Assembly in 2014. The Plan envisages scaling up kangaroo mother care (KMC) to 50% of babies weighing under 2000 g by 2020, and to 75% by 2025 [3].KMC was started in 1978 in Bogota, Columbia in response to inpatient overcrowdingand insufficient resources in neonatal intensive care units associated with highmorbidity and mortality among lowbirthweight (LBW) neonates [4]. KMC, as defined by Charpak, consists of three components, continuous skin-to-skin (STS) contact with the mother, exclusive breastfeeding, and early discharge from hospital in the kangarooposition with frequent home visits by health workers [4].

There is overwhelming evidence confirming KMC as an effective strategy to prevent mortality and morbidities in preterm and LBW babies in low, middle, and high-income countries [5–7]. KMC reduces rates of hypoglycaemia and neonatal infections and shortens the length of hospitalstay. KMC can be delivered in low-resourced settings without the demands of technology or electrical power [5]. Other reported benefits of KMC include improvement of the family environment [8] and rapid recovery of parents after a traumatic delivery, as well as strengthening family bonds [9].

Though KMC has been field-tested and found effective at health facility level, implementation and scale-up has been a challenge in lowincome countries, including Ethiopia. Several authors have reviewed these challenges at the level of health professionals, mothers and families [10–12]. A systematic review of 103 articles (27 from Sub-Saharan and North Africa) revealed resource.

and socio-cultural related factors, as well as a lack of clear guidelines and training for health care providers as barriers to KMC uptake [13]. Another study described fear, stigma, shame, guilt or anxiety relating to having a preterm infant or not wanting the baby, as a barrier to KMCimplementation [9]. Studies from highincomecountries have shown that caregivers who participate in giving good care of their newborn (made their newborns sleep longer, less anxious, more restful, more willing to breastfeed, and happier) are more likely to give skin-to-skin (STS) care [14–16].

Reactions from familyand communitymembers, including gender role and gender expectations, can also serve as either enabler if supportive or barriers if critical [17–19]. Another study underlined the negative influence of communication of mothers-in-lawand grandmothers on KMC uptake [19]. The same applies to the approach of health care workers, who could have a potential supporting [17, 18] or discouraging effect [18, 20]. A literature reported that keeping privacy of the mother make her feel respected and supportedby health professionals and found to be enablers of maternal KMC practice [21].

Although there is a body of literature on KMC, there is an evidence gap in Ethiopia regarding the barriers to and enablers of scaling up KMC at both health facility and community levels. KMC was first introduced in Ethiopia in 1996 at the Black Lion Hospital. Since then, KMC services have been expanded to other hospitals and health facilities at all levels. Recently, KMC was included in a series of policy documents issued by the Federal Ministry of Health the Newborn and Child Survival Strategy 2015–2020 [22]. Based on the assessment conducted in 2014 and 2015 in 6 hospitals and health centres across Ethiopia only 14% of babies born at the surveyed hospitals who weighed less than 2000 g were documented as enrolled into KMC [23]. In a recent study, based on the data from the 2016 national Emergency Obstetric and Newborn Care assessment which contains data on all health facilities providing delivery care services in Ethiopia, KMC was initiated for only 46.4% of all LBW babies [24].

This formative research, which is part of a large implementation research project undertaken in India and Ethiopia, was conducted in Southern Ethiopia and aimed to scale up KMC by identifying local barriers to KMC implementation in order to devise a refined model to deliver KMC across the community to facility continuum.

## Methods

A formative research study was conducted in Sidama district, southern Ethiopia to investigate the barriers to KMC practices. The study used a qualitative explorative approach that involved participants from health service providers and community members. The health service providers and community members approached directly through health institutions’ focal persons and community health workers, respectively. Twenty-four in-depth interviews (IDIs) and fourteen focus group discussions (FGDs) were carried out. A total of 144 purposively recruited study participants took part in IDIs and FGDs [25]. The study catchment area included 30 health centres and 85 health posts that referred mothers and babies to Adare, Leku, and Yirgalem Hospitals as well as to the regional referral centre, Hawassa University Referral Hospital (Table 1). Similarly, community members who could provide useful information about their experiences of KMC and LBW babies were selected from the communities of study sites. There were no formal exclusion criteria to select participants.

**Table 1:** Study participants and interview/FGD guides

Participants		Type and number of FGD or IDI	Number of participants	Average age	Interview guides
Health care providers at health facilities	Nurses	4 FGDs	24		• What do we mean by KMC? What are the components/elements of KMC? • Barriers and facilitators for KMC
	Midwives	4 FGDs	24		
	Physicians	12 IDIs	12	29 years	
Community health workers	Health development army	1 FGD	12		• Are babies usually weighed in a facility? What about at home? • How long after birth are they usually weighed? • What do you think is the best care for an early baby/ small baby in the facility? • Do women receive home visits after deliver within X days? What are the main reasons families may not receive a visit? • For what reasons would a newborn be referred to a facility? What is the referral process? How well does the referral system work?
	Health extension workers (HEWs)	1FGD	12		
Community members	Mothers with a Small baby	12 IDIs	12	• Mothers-27 years • Small babies-24 days	• Was your baby weighed after delivery? • Were you told the weight? • How did you feel about the baby being born early? • What did you think of the health providers in the facility in terms of attitude towards you and your baby? • What was the experience of KMC being done for the baby? • What is the family and community support during KMC?
	Mothers with a < 2-year old child	2 FGDs	24		• How would families feel if a mother delivered small or preterm baby? • How would they feel if the baby was born on time but was very small? • What problems can early babies have? What about small babies? • Is anything special done for babies that are born early/ small babies? • Who makes decisions about what is done for small or early babies at the facility, what about at home?
	Fathers with a < 2-year old child	1 FGDs	12		• What do you think about your baby’s weight? • How was your baby cared for after birth? • What do you understand by KMC? • What was the experience of KMC being done for the baby? (If the father provided KMC, ask about his experience) • Did you encounter any difficulty? what were these?
	Grandmothers with < 2-year old grandchild	1 FGDs	12		
**Total participants = 144**					

Health extension workers (HEWs) According to the healthcare plan of the Ethiopian Federal Ministry of Health, HEWs are female health workers who are trained for 1 year in 16 health packages which are expected to improve prevention skills and behaviors within the household and at the health posts. Health development army, refers to a massive unpaid community health workforce intended to improve population health and modernize the country

Semi-structured open-ended IDI and FGD guides (Table 1) were developed in English and later translated into local languages (Amharic and Sidaamu-Afoo) to be used for data collection. IDIs and FGDs were conducted at nearby health institutions and few IDIs took place at home. The duration of the FGDs was between 60 and 120 min whereas IDIs lasted between 45 and 60 minutes and all were audio recorded. The interviews and discussion process lasted until saturation of the ideas being discussed [26]. The IDIs and FGDs were conducted by 1 female and 2 male trained data collectors with 1st degree and above in health profession, and a good experience in qualitative data collection, and who are fluent in both Amharic and Sidamu-Afoo. Two data collectors with a Moderator were assigned to conduct each FGD, and similarly 1 data collector with a facilitator were assigned to run the in-depth interview. The FGD moderator and in-depth interview facilitator were with 2nd degree in reproductive health and a good experience in qualitative research. Additionally, some freedom of probes was allowed for deeper understanding, and notes were taken during the interview to record verbal cues.

The study applied a grounded theory approach [27] using systematic examination, to identify unanticipated phenomena and influences, to analyse and extract emerging themes from the data, and to generate new “grounded” theories for later development of a model for KMC. Ongoing data analysis took place throughout the study, in the process data were transcribed and translated by data collectors, and subsequently codes and themes were developed by investigation team. Data analysis was carried out manually. Early involvement in the analysis phase was helpful in facilitating back and forth development of themes and data collection, and in directing subsequent data collection towards sources that were more useful in constructing the best model.

Several steps were taken to reinforce credibility: we included participants from different districts; field notes taken during IDIs and FGDs were used in the analysis; and three of the study investigators participated in the data analysis [25]. Similarly, to satisfy the prerequisites for transferability, we provided descriptions of the settings, participants, inclusion and exclusion criteria, interview procedures and findings.

Ethical approval was obtained from Institutional Review Board at College of Medicine and Health Sciences, Hawassa University. Participants who were literate were provided with written information about the aims of the study, its benefits and confidentiality, and written consent was obtained from these participants. For those participants who were illiterate, the purpose of the study, its benefits, and confidentiality were explained by the interviewer or moderator and then verbal consent was obtained.

## Results

A total of 24 in-depth interviews and 14 FGDs were conducted. Twelve physicians (General practitioners and Paediatricians by profession) and 12 mothers with small babies participated in in-depth interviews. The average age of physicians was 29 years, and the average age of mothers and small babies was 27 years and 24 days, respectively. Majority of the mothers of small babies were married. Regarding FGD, 120 participants including health care providers (nurses, midwives, community health workers) and community members (mothers, fathers and grandmothers) had participated (Table 1). The formative research findings are presented and discussed in five different themes: missed opportunities in identification of preterm and LBW babies; poor referral system of preterm and LBW babies; community’s perceptions towards preterm and LBW babies; traditional care for preterm and LBW babies and challenges in KMC initiation and continuation.

### Missed opportunities in identification of preterm and LBW babies

In the study area, narratives of health care providers, mothers and community members indicated a high prevalence of home delivery. Some commonly mentioned reasons for not giving birth at health care facilities were a perceived lack of privacy, lack of awareness of institutional delivery, distrust in institutional delivery as a result of previous bad experience, negative attitude towards male delivery attendants, and grandmothers’ and elders’ influences. Moreover, mothers’ highly value traditional practices and ceremonies that take place at home during labour, during birth and after birth.

*“Some mothers may prefer home delivery because they believe that there is good progress of contraction* [labour] *at home due to good/warm temperature. But if the labour is at facility, the mother may be exposed to cold temperature and this may slow the contraction ... In addition, elder mothers’ and fathers’ influence (elders’ pressure) on mothers’ choice, by telling their past bad experiences of facility delivery.”* (FGD with health extension workers, Leku community)

The preference for home delivery is considered a challenge to identifying preterm and LBW babies in the study area. The narratives and accounts of most participants indicated that there is no practice of weighing babies born at home - instead mothers usually use traditional descriptions of size like: “*my baby is ‘fat’ or ‘medium’ or ‘thin’”.* Preterm and LBW babies are often identified seven days after the delivery when health extension workers visit mothers for postnatal care. Otherwise, babies born at home are weighed when they visit health facilities.

*“Most of the time, home delivered babies cannot get a birth weight measurement if they do not come to the hospital or health centre. Families may not know the benefits of bringing home born babies to a health facility.”* (IDI with health care provider)

Knowledge of the cut-off point for LBW seems to vary across health workers; < 1500 g,< 2000 g or < 2500 g. Additional gaps in accurate measurement and recording of birth weights were identified by health care providers. As a result, variations and inconsistencies in weighing babies at birth may occur in delivery and neonatal wards. Work overload, poor motivation, and negligence were among the reasons mentioned for missed opportunities in the identification of LBW babies. The type of weighing scale used was frequently mentioned as a challenge.

*“Currently we are using a non-standardized type of weighing scale which makes our work difficult. We are using a kilogram scale ... Generally, our scales have accuracy problems.”* (IDI with health care provider)

### Poor referral system of preterm and LBW babies

In general, there is limited attention to preterm and LBW babies during referral, whether within facilities or to facilities. Poor access to ambulance services and timely transfer to facilities for newborn care are major problems for mothers with small or preterm babies. Even in areas where ambulance services are available, there are delays in accessing newborn care services at KMC hospitals, and the services are patchy. Moreover, there is often poor documentation and lack of support from health care workers during the referral process for a small or preterm baby.

*“The referral system is not fast. Neonates who require early referrals do not access newborn care in a timely fashion. Some health centres refer babies* [to KMC hospitals] *24 hours after their arrival/delivery. During referrals, babies are not sent with proper documents and referral forms. Some times when documents are sent along, the medical treatment given to the baby is not included in the report. Health providers only write patient* [preterm/LBW babies] *names and reasons for referral. Some babies are taken to hospitals without an accompanying health provider ... … babies come only with an ambulance driver and so the referral system is not up to standard.”* (IDI with health care provider)

In the study, the absence of ambulance service for newborn care was identified as a major constraint that delays the referral of preterm and LBW babies. For this reason, small babies often come to the hospital by public transport or other means of transportation such as motor bicycle. As a result, babies are exposed to infection and hypothermia or, at worst, babies may die in transit. Even in the presence of an ambulance service, there is service inequity that may favour relatives or friends of district leaders.

*“... … somebabies come with an ambulance, and others do not. Most of the time the ambulance service is not available during the night shift. The family often use either public transport or motor cycle. As a result, most babies are exposed to a cold environment and some may even die. The ambulance service favours families if they have good relationships with district officials and health workers. Poor families and those who have no influencewith these officials find it hard to use the service”*FGD with health care provider)

### Community’s perceptions of preterm and LBW babies

Some study participants reported a belief that birth of a preterm or LBW baby was due to a *“curse of the day and disobedience of mainly mothers, family members and even kinship members to God’s command”.* We observed a wide range of local idioms and memes used to describe preterm and LBW babies. Local narratives included *“sick babies”*, and*“rat-sized babies”*. There were fathers who felt ashamed of having preterm or LBW babies, and who in turn put the blame on mothers for having those babies.

*“Family members experience deep grief and distress when a mother gives birth to a preterm/LBW baby; they* [family members (fathers, grandmothers and relatives)]*do not want to talk about it to neighbours. Mothers who give birth to preterm babies stay at home for a long time (up to six months) and avoid social contacts.”* (FGD with father of less than 2-year old child)

In addition, there is complete disapproval and denial of the survival of preterm and LBW babies. Sometimes even if they survive and grow to adulthood, the community attaches derogatory terms and stigma to them like *“s/he is a preterm person”* and *“this person is a 7-or 8-month man* [woman]”. Mothers described their experience of giving birth to a preterm baby as distressing, anxious, and stressful. Mothers also described such a moment as difficult, fearful, and full of doubt, especially when they think of their baby’s chance of survival compared to a full-term baby. Family members’ and the community’s perceptions of survival of small babies were not helpful, worsening mothers’ fears.

*“When I gave birth to a 7-month old baby in this hospital, I was distressed because many people were telling me that he could not grow and he might die. At the time I was unhappy, but now my baby is growing and sucking/feeding well at the breast.”* (FGD with mother of a small baby, now an under 2-year old child)

### Traditional care for preterm and LBW babies

There was poor community awareness of the importance and benefits of up to date care for small babies. Consequently, traditional newborn practices are used to protect the baby from illness and to maintain body temperature. In this community, *“evil eye”* is considered a very serious issue for the newborn and mother as well. To protect the mother and baby from the evil eye spirit, they are usually kept in a dark separate room (bedroom). Some mothers even cover their babies’ faces with plant leaves to avoid the gaze of visiting guests.

*“... … the mother covers the face of the baby with Bisana* [macrostachyus] *leaf in order to protect the baby from an attack by any evil spirit.”* (FGD with health care workers)

Besides, using baby clothes (such as hat, socks and cotton cloths) some families use “charcoal and fire” to keep preterm babies warm usually placing the charcoal close to (next to) the baby’s bed.

Additionally, parents feed a traditional herb soup *“hamessa”* or *“abish”*(fenugreek seed) and butter after homebirth to increase the baby’s weight, and they also believe that massaging the baby with butter strengthens and cleanses and *“and protects the baby from poliomyelitis and avoids abdominal cramp”.*

*“My family members didn’t know the importance of modern preterm/LBW care services, so they still challenge me. They tell me that the baby can grow at home by keeping his body warm and dressing the baby in cotton made clothes. They even encourage us to go home from the hospitals*.*”* **(**FGD with mother of a less than 2-year child)

*“Small/preterm/LBW babies are given “hamessa” and butter, which have no nutritional and medicinal value for them. It’s wrongly believed as being important to build and increase baby’s weight very quickly*... … ” (IDI with health care provider)

### Challenges in KMC initiation and continuation

Barriers to KMC practice at community level fell in two domains. Firstly, there were gaps in KMC knowledge, attitude, and practices among parents of preterm and LBW babies. Secondly, there were socio-economic, cultural and structural barriers identified in the community. Specific barriers to KMC implementation included the community’s beliefs, the value placed on preterm and LBW babies, the socioeconomic status of families, the acceptance of KMC by health professionals and their motivation, and the adequacy of essential supplies in health facilities. Study participants highlighted that*“mothers didn’t know anything about the KMC service when they first arrived at the hospital”*. Others reported that *“mothers and family members do not accept the service immediately, because of lack of awareness and information; they also refuse to practice the KMC service as they believe and consider that preterm/LBW babies do not survive, grow and develop like normal babies”*. It therefore takes some time for mothers and family members to accept and practice KMC effectively. A further challenge is an unreal expectation of mothers regarding their babies’ progress. As a consequence, they may become impatient, wishing to leave the facility before discharge criteria are fulfilled.

*“When they arrive at KMC ward most mothers are not aware of the KMC service … … It takes some time, and usually the first two days are challenging to convince and get mothers on KMC … … once they are counselled, they want to do it effectively within two or three days of admission … … Another challenge is, mothers are not happy staying for a long time in the facility and this might also be manifested bypoor weight gain in the baby”.* (IDI with health care provider)

Even if the mother is willing to accept the KMC practice, cultural barriers exist. For example, male dominance in a patriarchal society may override the mother’s acceptance and practice of KMC, as duration of hospital stay may be often determined by the father’s decision-making. Fathers’ and husbands’ willingness and commitment may depend on a variety of factors including male sex preference and number of children already at home. If the current small baby is a girl, or if they perceive that they have “enough” number of children, their acceptance and practice is impacted. Therefore, mothers often follow their husbands’ decisions with respect to staying in hospital for KMC. Moreover, participants expressed that the challenges seem to sum up to the community’s lack of exposure and experience with respect to KMC.

*“If the community has a good awareness and prior information about KMC practice, they would usually accept or approve it. On the other hand, if the community has no exposure to KMC, they may disapprove or refuse to accept it.”* (FGD with mother with young children less than 2 years of age)

Of importance, long distances to hospitals providing KMC, “high” cost for transport, charges for bed services, catering services, and drug expenses, and inability to buy KMC clotheswere financial hindrances to the utilization of KMC services.

*“Some mothers do not come with full materials or clothes... … They come to the hospital without a complete set of baby’s clothes such as hat and socks because they are poor and cannot afford to buy ... … So, the hospital has to supply all the required materials and clothes.”* (FGD with health care provider)

Similarly, numerous health system-related barriers in KMC wards and hospitals that limit access to quality KMC were identified, for example, lack of proper KMC infrastructure. Some hospitals lack a separate KMC ward to accommodate the privacy of mothers and their families and to promote the safety of small babies. Health care providers outlined that resource-related constraints appear to be substantial barriers to KMC practice.

*“We can't give enough KMC here as it is expected... … there are problems here* [Hospital]*, like shortage of beds, and because of that we sometimes send preterm/LBW babies to NICU, even though they are entitled to get KMC... thus, the shortage of beds couldn’t allow them* [babies] *to get proper KMC... … currently there are four beds in one room.”* (FGD with health care provider)

Regarding discharge of babies from KMC units, narratives and accounts of health care providers clearly depicted *“inconsistency and lack of uniform KMC discharge practice”*. Health care workers explained that hospital stay ranged from 7 to 20 days and the criterion for discharge was a target weight of 1600 to 1700 g. Some participants mentioned baby’s feeding progress as an additional criterion for discharge.

*“We have discharge criteria which are not similar for all babies, and usually we depend on the progress of the cases, some stay for 7days, some 20. We usually discharge preterm babies when they turn 37 weeks age and above and gain body weight up to 1600 or 1700grams...”* (IDI with health care provider)

KMC continuation after discharge (continue KMC at home) is another level care where family members and community health workers’ support are very crucial until the baby gain his/her normal weight. However, family support seems to be huge challenge for the mother to continue KMC at home, and to the worse the community attitude toward KMC appear to be very discouraging for majority of the mothers.

“ *… … ..I have given this care all the 11 days since discharge. I give the care 6-8 times a day where it stays for about 2 hours continuously. The care is more frequent during the day hours.” (IDI with mother of preterm/LBW baby)*

“ … ……… *There is no one who gives KMC in turn when I get tired. This is the most difficult problem I am facing right now”(IDI with mother of preterm/LBW baby)*

*“…. My villages feel bad when they come to visit me while I am giving skin to skin care for the baby….” (IDI with mother of preterm/LBW baby)*

Moreover, the expected visit from community health workers,(HEWs) give an impression that there is poor or lack of follow up during Postnata care.

“…*…. Nobody visits me after I got discharged from hospital…” (IDI with mother of preterm/LBW baby)*

## Discussion

This formative research study sought to identify the barriers to KMC implementation in rural and urban hospitals and community settings in Southern Ethiopia. FGDs and IDIs of health care providers, health care recipients, and family and community members were carried out. Several themes emerged:missed opportunities in identification of preterm and LBW babies; poor referral system of preterm and LBW babies; community’s perceptions towards preterm and LBW babies; traditional care for preterm and LBW babiesand challenges in KMC initiation and continuation.

. In each theme context, we identified many specific and modifiable barriers to KMC services, acceptance and practice. Finally, a model was developed to address barriers by proposing activities for KMC scale-up by enhancing its acceptance and practice.

Early identification of preterm and LBW babies is confounded by the persistence and high prevalence of home delivery leading to late recognition of those who would benefit from KMC. In keeping with other similar studies, there appears to be a lack of awareness in the community on the benefits of institutional delivery, and a preference for home birth in order to facilitate traditional ceremonies that take place at home [8, 9]. Ironically, identification of LBW babies may also be a challenge in health facilities due to inconsistency in measurement. Inconsistency was primarily ascribed to substandard weighing scales but human factors, such as lax weighing practices and work overload, were suggested as contributors.

Preterm and LBW babies are at greater risk of death than their full term and normal birth weight counterparts. Following identification, the next step is to transfer them (and their mothers) to an appropriate facility, where acute care and/or KMC can be provided. An inadequate referral system is a barrier to safe and timely access. To save lives and improve outcomes, a well-functioning referral system is mandatory; this should be accompanied by appropriate referral and transfer infrastructure and continued professional support.

Mothers’ acceptance and practice of KMC is inextricably linked with community acceptance and support and other socioeconomic factors. Specific contributors are the attitudes and support of fathers and grandparents. Poor community awareness and misconceptions regarding the causes, outcomes and survival of preterm and LBW babies were common factors. In addition, male dominance in decision-making (often discouraging KMC and hospital admission), and male sex preference greatly impacted mothers’ acceptance and practice of KMC. Mothers’ own stress and fear of having a preterm or LBW infant may influence their beliefs about survival and KMC care practice. Other studies have shown that fear, stigma, shame, guilt or anxiety about having a preterm infant were barriers to KMC initiation [9].

Studies suggest that many fathers feel that childcare should be a maternal role [19, 28]. However, this societal norm has an unpredictable effect on mothers’ decisions making regarding continuing KMC practice in a health facility. It appears that fathers’ decision-making authority and family members’ reactions may serve as enablers (if supportive) [17, 18] or barriers (if critical) [17, 19].Studies conducted in high [15, 29] as well as in low and middleincome countries [16] confirmed that fathers seem to need cultural support, but once they agreed, they could be very supportive to their partners, even performing skin-to-skin care themselves. Therefore, including fathers in counselling sessions is essential for good decision-making and support. Grandmothers are very close family members and they are actively engaged, especially in labour and early childcare practices. They have a huge influence in society, although there is preference for traditional childcare practices over modern health care practices. Involving them in the counselling sessions is something that should not be disregarded.

Mothers’ awareness of KMC was an important area highlighted by participants. Mothers did not know anything about KMC services when they arrived at the hospital. Consequently, in the first few days after birth, it was challenging to convince them to practice KMC. They were reluctant to remain in hospital for days because they had an expectation of overnight progress for their babies. This expectation may be due to poor counselling or lack of counselling materials. On the other hand, health workers’ knowledge and positive acceptance of KMC seem to be consistent, and an opportunity for improved counselling. One study reported that mothers were less likely to accept KMC if healthcare workers could not clearly explain its benefits. Parents reported that they were simply told to perform KMC without explanation of why, or how to do so, and expressed a feeling that KMC was forced upon them by caregivers [30]. Training KMC unit staff in counselling with supportive audio-visual aids would be an important step towards mothers’ and families’ commitment to KMC.

Regarding facilities, most participants revealed that long distances to KMC hospitals, “high” cost for transport, payment for bed services, catering services, and medicine expenses, and inability to buy KMC clothes were major financial barriers to KMC uptake. Similar conclusions were drawn in the KMC inpatient ward of a tertiary hospital in Malawi: mothers whose children died reported that a long distance to the health facility or shortage of money for transport expenses were reasons for not coming to the hospital [31].

Structurally, there was a lack of private space or enough space for mothers to perform KMC and to stay many days in hospital with their babies. The provision of private spaces, a quiet atmosphere, and dedicated resources could promote the acceptance and uptake of KMC [20]. Privacy screens or private rooms allow separating the family from hospital staff and other patients and offered a quieter atmosphere for mothers to practice KMC [30, 32]. Hospitals in implementation sites need support to establish dedicated KMC units that are mother-, baby- and family-friendly. Lastly, establish early contact after discharge with the community health workers, and create supportive home environment for the mother to continue effective KMC at home would be very helpful to see the baby’s final outcome.

To scale up KMC by enhancing its acceptance and practice, a model was developed that show activities at three different levels in order to overcome barriers, Table 2. The first level is pre-facility level which helps to reach out to community and primary health care units to identify all pregnancies and births at home and in health centres, and early referral of preterm/LBW to get KMC. The desired outcome at this level is the weighing of all newborns, and linking eligible babies to the health institution where KMC is available through the referral system.

**Table 2:** Model to scale up KMC

Service level	Desired outcomes	Where	Interventions				PLT
			Intended outcomes in the current health system		Planned activities to be done by the IST		
**Pre**-**facility**	• All pregnancies identified, registered and followed at regular intervals All home and HC births identified • Weigh all home and HC births delivered in the catchment areas • Refer all babies who are < 2000 g from HC and home • All referred babies access the referral site	Community and PHCU	• Favourable community awareness regarding facility delivery and care of small babies including KMC • KMC education during ANC visits • Established register of all pregnant mothers at health posts • Active reporting of home births by HDAs and HEWs • HEWs weigh all babies delivered at home (within 24 h) • Well-functioning referral system for small babies born at home • Proper counselling of mothers and family on KMC and small baby care during referral • Referral to facilities facilitated	Supportive supervision to lower tier health facilities regarding the referral, identification and referral	• Organize (with the RHB) community sensitization forums in public gatherings • Use PHCU meetings to pass KMC information on to HEWs and HDAs • Train HDAs and HEWs on the advantages of KMC and counselling on referral • Support HEWs through training and supervision to register pregnant mothers at health posts more actively • Establish a communication channel for HEWs with HDAs, 1–5 networks and pregnant mothers for birth notification • Avail and validate portable weighing scales (also train in their use). • Develop *aids* for referral and communication	Supporting integration of KMC mentoring and supervision in the existing system	Continuously assesses the barriers and enablers for KMC implementation at the catchment areas and presents its findings to the IST every two weeks.
**Facility**	• Initiation of KMC for all babies < 2000 g (in-born and referral) as soon as they fulfil eligibility criteria( • Continue safe implementation of KMC at facilities • Build women’s confidence and capacity to continue KMC at home	L & D	• All babies weighed properly • Babies below 2000 g referred to NICU/ KMC Unit (KMCU) of the district hospitals Skin-to-skin initiated within the first 1 h for all births		• Train and coach midwives on ECSB/KMC and proper weighing and calibration (and provide scales if none) Support hospitals to establish STS for all babies with in 1 h through training, mentoring and supervision		
		NICU	Babies who are below 2000 g actively transferred to the KMCU if they are stable; initiate KMC for sick babies as soon as they are stable		Training (including practical) and coaching on counselling of mothers and other family members while referring the baby to the KMC unit		
		KMC Unit	• KMCU established and renovated at hospitals • Mothers and family members educated on how to practice KMC • KMC practiced following the guidelines and developed follow up tools All mothers and family counselled on advantages of KMC		• Support hospitals in establishing dedicated KMC units • Provide dolls to KMC staff and train on how to use them for family training • Train and coach KMC staff on counselling mothers and family on KMC and caring for small babies KMC/ECSB training		
**Post**- **facility**	• Establish early contact after discharge • Support mothers to continue KMC at home	Post discharge PNC	• Addresses for all mothers discharged from the KMC units recorded • Strong linkage between mothers who are discharged, and community health workers established • HEWs do PNCs at 1,3,7 days after discharge and check on KMC practice, mother and baby health		• Update KMC registers (to include address for PNC tracking) • Develop and implement a communication system between hospitals (mentors) and CHWs (mentors call HEWs when a baby is discharged and follows up). • Calling mothers after discharge • Develop and implement PNC tracking cards for HEWs (with addresses of a contact person)		

Abbreviations: *PHCU* primary healthcare unit, *HEW* health extension worker, *RHB* regional health bureau, *HAD* health development army, *1–5 network* one community model family leading 4 others, *L and D* Labour and delivery, *NICU* newborn care unit, *KMC* kangaroo mother care, *ECSB* essential care for small babies, *PNC* Postnatal care

The second level is at health facility level where KMC services are provided. At this level, activities are proposed to ensure all eligible small babies, both inborn and referred, start and continue KMC.

The third level is post facility, where the aims to establish early contact after discharge and support the mother to continue KMC at home is addressed. Hence, this model proposes enablers in order to establish strong linkage between discharged mothers and community health workers (Health extension workers) who are responsible for providing home visits that support ongoing KMC as part of a KMC package.

Some limitations of this study should be mentioned. Demographic data of participants both in in-depth interviews and FGDs were partly missed during the data collection. Hence, the socio-demographic characteristics of in-depth interview participants both physicians and mothers of small babies presented is not complete. This might have a minor influence on the data but it did not affect the overall quality.

However,FGD grouping for health care providers were prearranged based on their specific profession such as FGD among nurses, midwives, and community health workers. Similarly, the FGD grouping among community members were prearranged based on their relationship to the baby such as FGD among mothers, fathers and grandmothers however other socio-demographic characteristics were not considered as eligibility criteria to be a FGD informant.

The data related to fathers’ support for mothers was rather weak. There is a deep cultural belief that mothers should not talk badly about their partners. Especially, we experienced that disclosing their partners’ wrong perception and poor practice of child care in front of other mothers in a FGD was a big deal for mothers. Most of the mothers preferred to keep quiet during the discussion, or only gave very short responses by referring to the comments of others. However, this limitation was minimized by the in-depth interviews with mothers; these might have still resulted in limitation to uncover paternal roles in KMC. Due to the rich data we have on both barriers and enablers of KMC practice, we could not present all in one paper. Therefore, in the current manuscript, more weight was given to the barriers of KMC but not enabling factors, and this might give an impression of bias. However, data on enabling factors are attached as supplementary file until we produce another paper on enabler factors of KMC practice.

## Conclusion

In conclusion, this study adds new insights into the current literature describing barriers to and challenges of KMC practice. A model has been developed that could provide further direction for researchers, programmers, policy makers and health workers with respect to KMC. This approach could be a more effective way to scale up KMC.For KMC uptake and acceptance, identifying all small babies and providing mothers support and resources is a priority. Although KMC has been proven to be a life-saving and cost-effective strategy in the management of small babies and their mothers, its implementation and scale has been challenging. This study describes an all-inclusive model embracing a continuum of care that employs systematic linkages between community and facility and supports the needs of important stakeholders.

## Supplementary Information

**Additional file 1: Table S1.** Study participants and interview guides.

## Data Availability

The data sets used in this manuscript is available with the corresponding author and will be made available up on reasonable request.

## Abbreviations

FGD: Focus Group Discussion
IDI: In-depth Interview.
KMC: Kangaroo Mother Care.
LBW: Low Birth Weight.
SSS: Skin-to-skin care.
HC: Health care.
HDA: Health Development Army.
HEW: Health Extension Worker.
