# Supplementary material for: Barriers for kangaroo mother care (KMC) acceptance, and practices in southern Ethiopia: a model for scaling up uptake and adherence using qualitative study
Source: BMC Pregnancy Childbirth. 2021 Jan 7;21:25. doi: 10.1186/s12884-020-03409-6 (PMC7789316; doi:10.1186/s12884-020-03409-6)
Supplement: Supplementary file 1 — Additional file 1: Table S1. Study participants and interview guides. [file 12884_2020_3409_MOESM1_ESM.docx]

| **Table 1: Study participants and interview/FGD guides** | | | | | |
| --- | --- | --- | --- | --- | --- |
| **Participants** | | **Type and number of FGD or IDI** | **Number of participants** | **Average age** | **Interview guides** |
| Health care providers at health facilities | Nurses | 4 FGDs | 24 |  | - What do we mean by KMC? What are the components/elements of KMC? - Barriers and facilitators for KMC |
|  | Midwives | 4 FGDs | 24 |  |  |
|  | Physicians | 12 IDIs | 12 | 29 years |  |
| Community health workers | Health development army | 1 FGD | 12 |  | - Are babies usually weighed in a facility? What about at home? - How long after birth are they usually weighed? - What do you think is the best care for an early baby/ small baby in the facility? - Do women receive home visits after deliver within X days? What are the main reasons families may not receive a visit? - For what reasons would a newborn be referred to a facility? What is the referral process? How well does the referral system work? |
|  | Health extension workers (HEWs) | 1FGD | 12 |  |  |
| Community members | Mothers with a  Small baby | 12 IDIs | 12 | - Mothers-27 years - Small babies-24 days | - Was your baby weighed after delivery? - Were you told the weight? - How did you feel about the baby being born early? - What did you think of the health providers in the facility in terms of attitude towards you and your baby? - What was the experience of KMC being done for the baby? - What is the family and community support during KMC? |
|  | Mothers with a  <2-yearold child | 2 FGDs | 24 |  | - How would families feel if a mother delivered small or preterm baby? - How would they feel if the baby was born on time but was very small? - What problems can early babies have? What about small babies? - Is anything special done for babies that are born early/ small babies? - Who makes decisions about what is done for small or early babies at the facility, what about at home? |
|  | Fathers with a  <2-yearold child | 1 FGDs | 12 |  | - What do you think about your baby’s weight - How was your baby cared for after birth? - What do you understand by KMC? - What was the experience of KMC being done for the baby? (If the father provided KMC, ask about his experience) - Did you encounter any difficulty; what were these |
|  | Grandmothers with  <2-year old grandchild | 1 FGDs | 12 |  |  |
| **Total participants= 144** | | | |  |  |
| *Health extension workers (HEWs) According to the healthcare plan of the Ethiopian Federal Ministry of Health, HEWs are female health workers who are trained for 1 year in 16 health packages who are expected to improve prevention skills and behaviors within the household and at the health posts . Health development army, refers to a massive unpaid community* health *workforce intended to improve population* health *and modernize the country.* | | | | | |
